# Epilepsy and Crime

**Published:** 1902-08-23

**Authors:** 


### EPILEPSY AND CRIME.

Dr. Spratling,[1] medical superintendent of the Craig Colony for epileptics, says that without attempting to formulate a definition applicable to all cases of so protean a malady as epilepsy, we may say that it is "a nervous disorder characterised by some impairment or loss of consciousness, coincident with some impairment or loss of the power of motor co-ordination, with or without convulsions, the paroxysms being generally abrupt in appearance and short and variable in duration." His object in so elaborating the matter is that the definition may recognise variations and degrees in its two most constant and prominent characteristics—the impairment of consciousness which is present in every epileptic attack and the impairment of motor co-ordination, which, however, may not be enough to be appreciated. It is to the impairment of consciousness that he specially directs attention. The effects of epilepsy upon the mind are, he says, temporary, prolonged, or permanent. The continuously insane epileptic is not usually liable to commit crime, his lunacy being marked enough to secure his restraint in an asylum. But even such an one, although insane, does not lose his epileptic identity ; he continues to have convulsions, and it is the rule for his insanity to become more marked just before his seizures, his irascibility and tendency to violence increasing sharply at such times and declining with the passing of these periods ; and although it is true that permanently insane epileptics are generally harmless because they are generally under restraint, these sudden outbursts of violence are full of danger in the case of those who are at home. The epileptic who is subject to prolonged periods of mental disturbance and yet not continuously insane is a great difficulty, for his epilepsy is not constant in type. He may have a grand mal seizure to-day and a petit mal to-morrow, and after either the one or the other he may continue in a condition of automatism for a time ranging from a few minutes to even weeks. He is fully able to go about as usual, to look after himself, and to do commonplace things it was his habit to do before the attack. Yet his mind is a blank, while his bodily actions go uninterruptedly on. A person in such a state could have no criminal intent. The same condition of automatism may occur independent of attacks that disturb motor co-ordination. A purely psychic epilepsy. The greatest medico-legal problem connected with epilepsy "is encountered in its purely psychical form, in those uncomplicated by any motor disturbance; that make no rude sign of their approach ; that sometimes last for hours, or days, or even weeks, and finally pass away as silent as they came." In such cases we encounter, says Dr. Spratling, a somewhat anomalous situation, for while the law recognises the absence of responsibility in insanity it fails to do so in epilepsy, and thus it happens that a man who commits a crime while in a state of psychic epilepsy or post-epileptic automatism must either be found guilty and made to suffer for his act or be acquitted and allowed to go free. In many cases, however, epilepsy and insanity are interchangeable terms and might well be so regarded. It was held, years ago, by a judge of the Supreme Court of Alabama that the true test of responsibility in cases of insanity is not merely the knowledge of right and wrong, but the power to refrain from the act, and "the application of a similar test in establishing responsibility in epilepsy would seem especially fitting and just."

[1] Journal of Nervous and Mental Disease, August.

